# Cisplatin-Induced Eosinophilic Pneumonia

**DOI:** 10.1155/2014/209732

**Published:** 2014-11-13

**Authors:** Hideharu Ideguchi, Keisuke Kojima, Susumu Hirosako, Hidenori Ichiyasu, Kazuhiko Fujii, Hirotsugu Kohrogi

**Affiliations:** Department of Respiratory Medicine, Kumamoto University Hospital, 1-1-1 Honjo, Chuo-ku, Kumamoto 860-8556, Japan

## Abstract

A 67-year-old man suffering from esophageal cancer was admitted to our hospital complaining of dyspnea and hypoxemia. He had been treated with cisplatin, docetaxel, and fluorouracil combined with radiotherapy. Chest computed tomography revealed bilateral ground-glass opacity, and bronchoalveolar lavage fluid showed increased eosinophils. Two episodes of transient eosinophilia in peripheral blood were observed after serial administration of anticancer drugs before the admission, and drug-induced lymphocyte stimulation test to cisplatin was positive. Thus cisplatin-induced eosinophilic pneumonia was suspected, and corticosteroid was effectively administered. To our knowledge, this is the first reported case of cisplatin-induced eosinophilic pneumonia.

## 1. Introduction

Cisplatin (CDDP) is one of the most common anticancer drugs and is approved for the treatment of many types of cancer. It inhibits DNA synthesis by the formation of DNA cross-links. CDDP-induced lung disease is unusual. Many types of drug, including anticonvulsants, antidepressants, nonsteroidal anti-inflammatory drugs, antihypertensives, and anticancer drugs, are well known to cause eosinophilic pneumonia. However, eosinophilic pneumonia induced by CDDP has not been reported.

## 2. Case Report

A 67-year-old man was admitted to our hospital with the complaint of severe dyspnea. Four months prior to admission, he had been diagnosed with esophageal cancer and treated with four cycles of chemotherapy with CDDP, docetaxel, and fluorouracil combined with radiotherapy (2 Gy/day × 30 days; total 60 Gy). Seven days previously, he had complained of dyspnea and fever, and a chest computed tomography scan demonstrated bilateral diffuse ground-glass opacity. He was diagnosed with community-acquired pneumonia and was treated with garenoxacin (400 mg once a day). In spite of the antibacterial therapy, the exertional dyspnea and oxygenation worsened.

**Figure 1:**
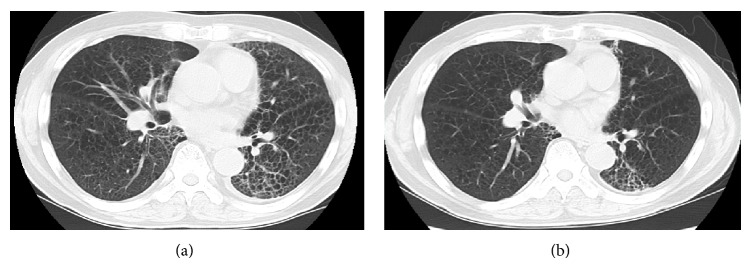
Chest CT scan on admission (a) shows bilateral ground-glass and reticular opacities. Fifteen days after the administration of corticosteroid, bilateral ground-glass opacities reduced (b).

Respiratory distress was shown with SpO_2_ of 87% on room air. Physical examination revealed rhonchi in the left upper lung and localized skin eruption due to radiotherapy at the precordium. Laboratory findings demonstrated an elevated peripheral eosinophil count of 1152 cells/*μ*L, C-reactive protein level of 6.88 mg/dL (normal range: <0.3 mg/dL), lactate dehydrogenase level of 312 U/L (112–213 U/L), and KL-6 level of 796 U/mL (<401 U/mL). The total IgE level was 137 IU/mL (<400 IU/mL). His chest radiograph showed bilateral ground-glass opacity compared with that observed four months previously. A chest computed tomography scan demonstrated bilateral ground-glass and reticular opacity (Figure 1(a)). Pulmonary function tests revealed impaired diffusion capacity, with 44.4% of diffusing capacity of the lung for carbon monoxide (%DLco).

**Figure 2:**
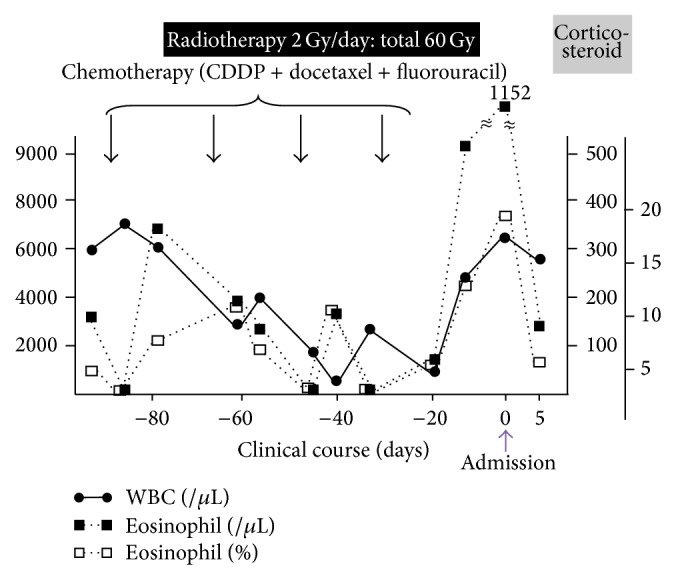
Clinical course. Note that percentage (open squares) and absolute number (closed squares) of peripheral eosinophil transiently increased just after the serial administration of anticancer drugs and it dramatically increased after the 4th course of the chemotherapy. Eosinophil dramatically decreased after the administration of corticosteroid.

Infectious diseases, malignancies, cardiovascular diseases, and collagen vascular diseases were carefully ruled out. Bronchoalveolar lavage fluid showed increased eosinophils (24.3%), consistent with eosinophilic pneumonia. Interestingly, episodes of transient eosinophilia were found after the serial administration of anticancer drugs, CDDP, docetaxel, and fluorouracil (Figure 2). Drug-induced lymphocyte stimulation test to CDDP was positive (stimulation index was 184%), while those to docetaxel (90%) and fluorouracil (77%) were negative. We therefore diagnosed the patient as having CDDP-induced eosinophilic pneumonia. We administered systemic corticosteroid, methylprednisolone, at 125 mg once a day for three days, followed by oral prednisolone (30 mg once a day). Fifteen days after the administration, the percentage of peripheral blood eosinophils decreased from 18.3% to 3.8%, and the increased opacity on chest computed tomography scan was improved (Figure 1(b)). PaO_2_ on room air was highly improved from 62.8 to 83.2 Torr. The dose of prednisolone was tapered over nine months to zero, and he had no recurrence.

## 3. Discussion

The diagnosis of drug-induced eosinophilic pneumonia essentially rests on the temporal association between exposure to an administered drug and the development of pulmonary infiltrates, although the mechanism involved is complex. In the present case, we first suspected CDDP, docetaxel, or fluorouracil as the causative drug because episodes of transient peripheral blood eosinophilia had been observed a few days after the administration of these anticancer drugs. In addition, positive results of drug-induced lymphocyte stimulation test to CDDP suggested that the most likely causative drug was CDDP.

The diagnosis of drug-induced lung disease involves exclusion of all other possible causes. In the present case, radiation pneumonitis was also suspected as the cause of the pulmonary infiltrates because the patient was treated with radiotherapy before the onset. In radiation pneumonitis, lymphocytes were shown to increase in the bronchoalveolar lavage fluid; in addition, a few case reports of radiation-induced eosinophilic pneumonia have been published [1]. Recently, it has been suggested that induction of some cytokines such as TNF*α* and IL-1*β* by irradiation increases the expression of an eosinophil chemoattractant, eotaxin, in the lung epithelial cells or that irradiation itself may increase the expression of eotaxin and its receptors [2, 3]. However, the mechanisms of eosinophilic pneumonia induced by radiation have not yet been established. In the present case, because the first episode of peripheral blood eosinophilia was observed before the radiotherapy, radiation pneumonitis induced-eosinophilia is not likely.

Many different mechanisms are involved in the initiation and propagation of drug-induced pneumonitis [4]. Drugs cause lung injury by direct toxicity or by triggering immunologic responses, but often this neat distinction is unclear by the combined effects of preexisting lung injury and the concurrent administration of several drugs. For drug-induced pneumonitis, a pattern of eosinophilic pneumonia is the most suggestive of triggering immunologic responses [5]. In the present case, episodes of transient peripheral blood eosinophilia just after serial chemotherapy and positive results of drug-induced lymphocyte stimulation test to CDDP were observed. Thus, we concluded that CDDP was the causative agent of the eosinophilic pneumonia. Here, we are the first to report a case of eosinophilic pneumonia induced by CDDP.
